# Mobile Phones: Reservoirs of Resistant Bacteria during the COVID-19 Pandemic in Abu Dhabi, United Arab Emirates

**DOI:** 10.3390/microorganisms11020523

**Published:** 2023-02-18

**Authors:** Kawthar Kayed, Ghalia Khoder, Joviana Farhat, Rose Ghemrawi

**Affiliations:** 1 College of Pharmacy, Al Ain University, Abu Dhabi P.O. Box 112612, United Arab Emirates; 2 AAU Health and Biomedical Research Center, Al Ain University, Abu Dhabi P.O. Box 112612, United Arab Emirates; 3 Department of Pharmaceutics and Pharmaceutical Technology, College of Pharmacy, University of Sharjah, Sharjah P.O. Box 27272, United Arab Emirates; 4 Research Institute for Medical and Health Sciences, University of Sharjah, Sharjah P.O. Box 27272, United Arab Emirates; 5 Department of Epidemiology and Population Health, College of Medicine and Health Sciences, Khalifa University, Abu Dhabi P.O. Box 127788, United Arab Emirates

**Keywords:** bacterial contamination, mobile phones, COVID-19 pandemic, antibiotic resistance, disinfection

## Abstract

Background: Mobile phones are excessively used even though microbes’ ability to survive on phone surfaces was confirmed. During the COVID-19 pandemic, heavy hygiene practices have been applied to mobile surfaces. Therefore, it is interesting to evaluate the emergence of antimicrobial-resistant bacteria on mobile phone surfaces. Methods: A random sampling technique was utilized on residents in Abu Dhabi, UAE between May and June 2021. A swab sample from each participant’s mobile phone was collected and transported to the microbiology laboratory for bacterial culture and antimicrobial susceptibility tests. Furthermore, a cross-sectional study was conducted via a self-administered questionnaire filled by participants. The questionnaire was used to collect sociodemographic data, phone frequency usage and cleaning methods. Results: One hundred two-sample swabs and data have been included in the study. The majority of participants (91.1%) reported cleaning their mobile phones with wipes and alcohol. However, 100% of participants had a mobile phone contaminated by bacteria such as *S. aureus, E. coli, Coagulase-negative staphylococci, Micrococcus, Bacillus, Streptococcus, Citrobacter, Proteus, Enterococcus, klebsiella, Pseudomonas* and *Actinobacteria*. Interestingly, most of these potentially pathogenic bacteria were found to be resistant to ampicillin, ceftazidime and cefotaxime. Conclusion: The continuous hand and mobile disinfectant have contributed to the emergence of resistant bacteria.

## 1. Introduction

Portable electronic devices, such as keyboards and smartphones, are regularly used everywhere and by everyone; however, most people are not aware that these devices can accumulate and transmit microorganisms [1,2,3,4].

More than 7.26 billion persons worldwide are mobile phone users, so roughly 91.69% of the population own a phone [5]. The number of smartphone users in the United Arab Emirates (UAE) exceeds 17.1 million [6]. Knowing that the UAE’s population in 2022 stands at 10.08 million, this means that some individuals own more than one smartphone. Despite high usage percentages, most people do not know that phones are potential vectors for the transmission of infections [3,4]. Human skin, wallets, bags and even shisha, especially in Arab countries, are significant sources of contamination for mobile phones [7,8].

Several studies conducted on cell phones have confirmed their contamination with microbes, such as *Escherichia coli (E. coli)*, *Coagulase-negative staphylococci (CoNS)*, *Staphylococcus aureus (S. aureus),* etc. [9]. Not only fecal–oral transmission and infected skin but also contaminated cell phones were found to be the main route by which pathogenic bacteria cause infections [8,10,11]. Approximately 80,000 to 180,000 infections could be avoided every year [12], especially by adequate hand hygiene and increased intensity of smartphone cleaning [13]. The COVID-19 pandemic has drawn public attention to improving personal hygiene measures [14]. The UAE government introduced several safety standards for individuals to follow amid the pandemic. Health authorities handled the situation efficiently, adopted an integrated strategy, implemented intensified awareness campaigns on public hygiene and made mandatory sterilization supplies in all public places [15].

According to WHO, antimicrobial resistance (AMR) is one of the threats to global health and 10 million people worldwide are predicted to die due to AMR by 2050 [16]. UAE residents were heavily using antimicrobial detergents during the COVID-19 pandemic [15], however, the level of AMR in Abu Dhabi, UAE was not evaluated. Therefore, our study aimed to isolate bacterial strains from mobile cell phone surfaces, identify them then assess their antimicrobial susceptibility pattern.

## 2. Materials and Methods

### 2.1. Ethical Considerations

The ethical approval of the study was taken from the research ethics committee (REC) of Al-Ain University (AAU-REC-B3, May 2021). Participation in the study was completely voluntary. Written informed consent was obtained from all participants.

### 2.2. Study Design

This cross-sectional study was conducted during the COVID-19 pandemic, between May and June 2021 in Abu Dhabi, UAE. Swab samples were collected from mobile phones of academic and admin staff working in Universities in Abu Dhabi. Before the collection of the swab samples from the surface of the mobile phones, participants were requested to fill in a questionnaire of two sections. Section one consisted of basic sociodemographic variables (age, gender, marital status, education level). In section two of the survey, we investigated mobile hygiene practices. Questions in this section were related to the possession of a smartphone or keypad, with or without cover, the mobile usage frequency at work, phone usage in bathrooms, frequency of hands washing, mobile phone storage at work (on the desk, in the drawer, in the pocket or in the bag), mobile phone usage by kids, husband or wife and if they cleaned their phones using wipes, alcohol or water. The questionnaire was filled by participants under the supervision of the investigator to ensure that the questions were clear, not ambiguous and understandable.

### 2.3. Inclusion and Exclusion Criteria

Females and males over the age of 18 years, residents of Abu Dhabi, UAE and working in a university in contact with students were included in the study. People with mental health issues and those who did not consent to participate were excluded from the study.

### 2.4. Sample Size and Sample Collection

One hundred and five individuals participated in this study, three of them did not give their consent so they were excluded. First, detailed information about the study was explained to the participants. Then, private mobile phones, used during work, were retrieved for microbiological testing, without any prior sanitization or purification. Consistency was ensured by performing all sampling by one investigator. To ensure proper sampling, both hands of the swab collector were cleaned using an alcohol-based hand sanitizer before swab collection. To prevent any potential cross-contamination, the collector was requested additionally to wear gloves and a mask. One hundred two samples were collected aseptically using a plain sterile swab. Sterile cotton swab (BROMED, USA), moistened with sterile normal saline solution (0.85%) (Pharmaceutical Solution Ind, UAE), was rotated over the screen, front, back and sides of mobile phones as these zones are most frequently in contact with fingers. Swabs were also rubbed on the outer surface of mobile phone covers. Swab samples were labelled carefully in accordance with the filled questionnaire and transported within one hour to the microbiology laboratory in aseptic and refrigerated conditions.

### 2.5. Bacterial Culture and Identification

Sampled mobile phone swabs were streaked onto nutrient agar (Sigma-Aldrich, Telangana, India). The inoculated plates were then incubated aerobically in an inverted position at 37 °C for 24 to 48 h for bacterial culture, identification and antimicrobial susceptibility testing. Plates were then observed for the presence of isolated colonies. Selected colonies were again sub-cultured on nutrient agar to isolate pure culture, followed by further identification and characterization on MacConkey agar (Sigma-Aldrich, St. Louis, MO, USA), Mannitol salt agar (Sigma-Aldrich, Telangana, India), and Eosin methylene blue agar (Sigma-Aldrich, Madrid, Spain) [17]. Isolated bacteria were identified by conventional microbiological methods using macroscopic examination based on colony morphology and microscopic examination using based on Gram-staining (HiMedia, Maharashtra, India). For further identification, various biochemical tests such as oxidase (Sigma-Aldrich, St. Louis, MO, USA), catalase (Sigma-Aldrich, St. Louis, MO, USA), coagulase (Sigma-Aldrich, Madrid, Spain), DNase (HiMedia, Maharashtra, India), methyl red (MilliporeSigma, Karnataka, India) Voges-Proskauer (MilliporeSigma, Karnataka, India), citrate (MilliporeSigma, Zug, Switzerland), and oxidative-fermentation (MilliporeSigma, Zug, Switzerland) tests were carried out on isolated bacterial colonies [17]. A non-inoculated culture media of all the used media were used as a negative control of the study.

### 2.6. Antimicrobial Susceptibility Test (AST)

Antibiotic susceptibility tests were performed using the disk-diffusion method based on the Clinical and Laboratory Standards Institute guidelines (CLSI, 2015) using Mueller Hinton agar (Sigma-Aldrich, India) [18]. From the pure bacterial culture grown overnight on nutrient agar, a bacterial suspension matching 0.5 McFarland standard (1.5 × 10^8^ cfu/mL) was prepared in nutrient broth. Mueller–Hinton agar plates were inoculated by the lawn culture method using a sterile cotton swab. The following antibiotic disks have been used: gentamicin (GN, 30 μg) (Bioanalyse, Turkey), ciprofloxacin (CIP, 30 μg) (Bioanalyse, Turkey), ceftazidime (CAZ, 30 μg) (Liofilchem, Italy), cefotaxime (CTX, 30 μg) (Liofilchem, Italy) and ampicillin (AM,10 μg) (Bioanalyse, Turkey). The choice of antibiotics was based on previous work performed by Khadka et al. and Rozario et al. [19,20]. Antibiotic sensitivity test results were confirmed by Kirby–Bauer disk diffusion method according to the Clinical and Laboratory Standards Institute (CLSI) guidelines.

## 3. Results

### 3.1. Participants’ Characteristics

One hundred and two participants from Abu Dhabi, UAE were included in the final analysis. Table 1 shows their sociodemographic characteristics and mobile hygiene practices. Forty-seven percent (*n* = 48) of participants were aged between 22 and 34, 31.4% (*n* = 32) were between 35 and 44, 21.5% (*n* = 22) were more than 45 years old. Fifty-three participants were male accounting for 52% of the studied sample, and 48% (*n* = 49) were female. The majority (66.7%) were married, among which 37.3% (*n* = 38) allowed their family to use their phones. Half of them had a bachelor’s degree, 17.6% (n18) a master’s degree and 32.4% (*n* = 33) a PhD. The majority of the respondents had a screen touch phone (98%, *n* = 100)) with a phone cover (75.5%, *n* = 77). The majority (66.7%, *n* = 68) used their phones during work more than six times per day, 73.5% (*n* = 75) used to keep them on their desks and 42.2% (*n* = 43) used their phones in the bathrooms. Interestingly, 40.2% (*n* = 41) washed their hands more than 11 times per day and 91.1% (*n* = 93) of participants cleaned their mobile phones using wipes (52.9%, *n* = 54), alcohol (34.3%, *n* = 35) or water (3.9%, *n* = 4).

### 3.2. Mobile Phone Contamination

**Figure 1:**
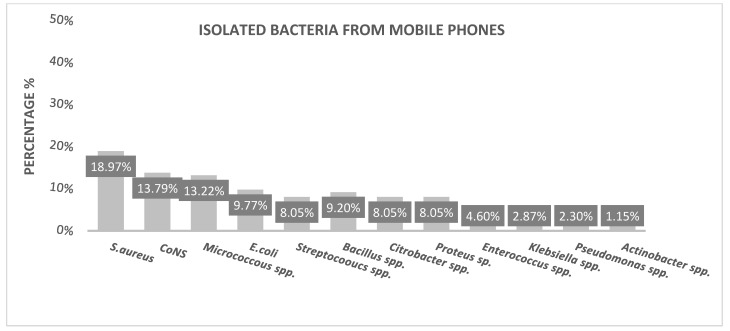
Mobile bacterial distribution profile. *S. aureus; CoNS; Micrococcus* species; *E. coli; Streptococcus* species, *Bacillus* species; *Citrobacter* species; *Proteus* species; *Enterococcus* species, *Klebsiella* species, *Pseudomonas* species, *Actinobacter* species.

A total of 100% of mobile phones were contaminated by bacteria. As shown in Figure 1, the most abundant isolates were *S. aureus* (18.97%), followed by *CoNS* (13.79%), *Micrococcus* spp. (13.22%), *E. coli* (9.77%), *Streptococcus* spp. (8.05%), *Bacillus* spp. (9.2%), *Citrobacter* spp. and *Proteus* spp. (8.05%), *Enterococcus* spp. (4.6%), *klebsiella* spp. (2.87%), *Pseudomonas* spp. (2.3%) and, finally, *Actinobacteria* spp. (1.15%).

### 3.3. Antimicrobial Susceptibility Pattern of Bacterial Isolates

The antimicrobial susceptibility profiles of the isolates are shown in Table 2. All of the isolated Gram-positive and Gram-negative microorganisms showed sensitivity to ciprofloxacin (100%). *S. aureus* was susceptible to cefotaxime (81.8%), gentamicin (96.9%) and ceftazidime (51.5%), while 20 isolated *S.aureus* showed resistance against ampicillin (60.6%). The susceptibility patterns of *CoNS* showed that they were sensitive to cefotaxime (83.3%), ampicillin (50%), gentamicin (100%) and ceftazidime (75%). Seventeen isolated *E. coli* showed sensitivity to gentamicin (100%), but 14, 9 and 11 of the isolated samples showed sensitivity to cefotaxime (82.3%), ampicillin (52.9%) and ceftazidime (64.7%), respectively. *Streptococcus* spp. and *Bacillus* spp. showed resistance against ampicillin (71.4%), (68.75%) and ceftazidime (64.3%), (62.5%), respectively. All 37 isolates of *Citrobacter* spp., *Proteus* spp., *Klebsiella* spp. and *Pseudomonas* spp. were tested in presence of cefotaxime, ampicillin, gentamicin and ceftazidime. Twenty-eight from the isolated *Citrobacter* spp. (78.5%), *Proteus* spp. (78.5%), *Klebsiella* spp. (60%), *Pseudomonas* spp. (75%) showed resistance, especially against ampicillin (Table 2). Twenty-three (13.22%) *Micrococcus* spp. were found, 20 isolates (86.9%) were sensitive to cefotaxime, 23 isolates (100%) were sensitive to gentamicin, 15 to ceftazidime and 13 were resistant to ampicillin (56.6%).

## 4. Discussion

One hundred and two participants from the emirates of Abu Dhabi in UAE were included in this study. The majority of the respondents used their phones during their work more than six times per day, and 42.2% used their phones in the bathrooms. These findings are not surprising since workers, even those in the healthcare system were found to use their phones as work aid and in the bathrooms [21].

Interestingly, 91.1% of respondents cleaned their mobile phones using either wipes (52.9%), alcohol (34.3%) or water (3.9%). Sampling was performed during the COVID-19 outbreak where frequent disinfection and sanitization of hands, touched objects and fomites was encouraged worldwide [22], and this pandemic correlated with an increase in disinfectant usage and consumption [23]. Furthermore, a previous study, evaluating the public perceptions to mitigate the spread of the COVID-19 pandemic in UAE, showed that the public response to the government-imposed preventive measures was robust; individuals in UAE were found to be very cautious against the virus [24].

Even though mobile phones were frequently cleaned, all of them were found to be contaminated by bacteria, such as *S. aureus*, *CoNS*, *E. coli*, *Bacillus* spp., *Streptococcus* spp., *Proteus* spp., *klebsiella* spp., *Pseudomonas* spp. and *Actinobacteria* spp. It is worth noting that the bacterial contamination degree found on mobile surfaces, and the type of identified bacteria differed between the surfaces that have been sampled (Supplementary Table S1). The type of identified bacteria is in concordance with other studies from 24 different countries such as Saudi Arabia, Turkey, France, Ghana, Egypt, India, Australia, Iran, South Korea and Poland [25]. However, more types were identified in our study, such as Micrococcus, Enterococcus and Citrobacter [11].

Our study revealed that *S. aureus* was the most commonly isolated organism where 18.97% of the tested samples have shown *S. aureus* growth. This is in line with previous work performed in Ethiopia [26] and Turkey [27]. In our study, *CoNS* was ranked second. However, the majority of other studies [28] showed that *CoNS* was the most common isolate.

Interestingly, most bacterial isolates were resistant to at least one antimicrobial agent, as shown in Supplementary Table S2. This can be explained by the fact that we found 91.1% of our participants cleaned their phones either using wipes, alcohol or water.

Antimicrobial resistance is a major concern to human health. Worldwide, 4.95 million deaths are associated with bacterial resistance to antimicrobials. *E. coli, S. aureus, Klebsiella* spp., *Streptococcus* spp., *Acinetobacter* spp. and *Pseudomonas* spp. are known to be leading pathogens for deaths associated with resistance [29]. In our study, they were all found to be resistant to ampicillin, ceftazidime and cefotaxime. High rates of ampicillin resistance were observed in UAE between 2014–2019 [30]. However, resistance to ceftazidime and cefotaxime was never found in UAE [31,32]. This can be explained by the increased use of antibacterial cleaning and hygiene products during the COVID-19 pandemic which is known to be a risk factor for microbial resistance [33].

In the future, in order to identify the metagenomic presence of micro-organisms on phones’ surfaces, DNA extraction followed by downstream next-generation sequencing shotgun microbial profiling will be performed. Mobile phones are also heavily used in healthcare settings [21] which may be a potential source of microbial dissemination. Therefore, it would be interesting to assess the contamination of mobile phones owned by hospital medical staff in Abu Dhabi, and to detect antibiotic resistance and virulent factors.

## 5. Limitations

- The use of a self-reported questionnaire may create some biases since respondents may offer biased self-estimation.
- Participants were not asked if they removed their phone covers before cleaning them, nor if they allowed their phones to dry after the cleaning process.
- The study did not address the effect of period variations.
- The percentage of participants over 45 is 21.5% (*n* = 22). However, the number of participants who were over 65 years old is unknown. Therefore, it is not possible to assess the unintended consequences of this AMR in the elderly population.

## 6. Conclusions

Our study was held during the COVID-19 pandemic which witnessed a remarkable change in people’s hygiene behavior. Therefore, we found that 91.1% of our study participants used to clean their mobile phones mostly with wipes and alcohol. However, these devices were contaminated with *S. aureus*, *CoNS*, *Micrococcus* spp., *E. coli*, *Bacillus* spp., *Streptococcus* spp., *Citrobacter* spp., *Proteus* spp., *Enterococcus* spp., *Klebsiella*, *Pseudomonas* spp. and *Actinobacteria* spp. Most of these potentially pathogenic bacteria were found to be resistant to ampicillin, ceftazidime, cefotaxime. Resistance to ampicillin was already found in UAE, but we are the first to find resistance to ceftazidime and cefotaxime among bacteria in Abu Dhabi. This shows that the increased use of wipes and alcohol during the COVID-19 pandemic may be linked to increased antimicrobial resistance.

## Figures and Tables

**Table 1:** Participants sociodemographic characteristics and mobile hygiene practices.

	Overall (*n* = 102)	
	*n*	%
Age		
22–34	48	47.1
35–44	32	31.4
≥45	22	21.5
Gender		
Male	53	52
Female	49	48
Marital status		
Single	34	33.3
Married	68	66.7
Education level		
Bachelor	51	50
Master	18	17.6
Doctorate	33	32.4
Phone type		
Screen Touch	100	98
Keypad	2	2
Phone cover		
Yes	77	75.5
No	25	24.5
Mobile usage frequency at work		
1–5 times per day	34	33.3
6–9 times per day	21	20.6
≥10 times per day	47	46.1
Phone usage in bathrooms		
Yes	43	42.2
No	59	57.8
Frequency of hands washing		
1–10 per day	61	59.8
11–20 per day	41	40.2
Mobile phone storage at work		
Desk	75	73.5
Drawer	4	3.9
Pocket	18	17.6
Bag	5	4.9
Mobile phone usage by kids, husband or wife		
Yes	38	37.3
No	64	62.7
Phone cleaning		
Wipes	54	52.9
Alcohol	35	34.3
Not applicable	9	8.8
Water	4	3.9

**Table 2:** Antimicrobial susceptibility profiles of bacterial isolates from mobile phones. Gentamicin (GN, 30 μg), Ciprofloxacin (CIP, 30 μg), Ceftazidime (CAZ, 30 μg), Cefotaxime (CTX, 30 μg) and Ampicillin (AM,10 μg).

Organism	Antimicrobial Susceptibility (%)					
	S/R	Ciprofloxacin	CTX	AM	GN	CAZ
S. *aureus* (*n* = 33)	S	100	81.8	39.3	96.9	51.5
	R	0	18.1	60.6	3.03	48.4
*CoNS* (*n* = 24)	S	100	83.3	50	100	75
	R	0	16.6	50	0	25
*E. coli* (*n* = 17)	S	100	82.3	52.9	100	64.7
	R	0	17.6	47.05	0	35.3
*Streptococcus* spp. (*n* = 14)	S	100	85.7	28.5	100	35.7
	R	0	14.2	71.4	0	64.3
*Bacillus* spp. (*n* = 16)	S	100	75	31.25	100	37.5
	R	0	25	68.75	0	62.5
*Citrobacter* spp. (*n* = 14)	S	100	71.4	21.4	100	57.1
	R	0	28.5	78.5	0	42.8
*Proteus* spp. (*n* = 14)	S	100	71.4	21.4	100	57.1
	R	0	28.5	78.5	0	42.9
*Enterococcus* spp. (*n* = 8)	S	100	100	50	100	62.5
	R	0	0	50	0	37.5
*Klebsiella* spp. (*n* = 5)	S	100	60	40	100	40
	R	0	40	60	0	60
*Pseudomonas* spp. (*n* = 4)	S	100	50	25	100	25
	R	0	50	75	0	75
*Micrococcus* (*n* = 23)	S	100	86.9	43.47	100	65.2
	R	0	13.04	56.5	0	34.8
*Actinobacteria* (*n* = 2)	S	100	100	50	100	100
	R	0	0	50	0	0

## Data Availability

The data supporting the results are available upon request.

## Supplementary Materials

The following supporting information can be downloaded at: https://www.mdpi.com/article/10.3390/microorganisms11020523/s1, Table S1: Identified bacteria on participants’ mobile surfaces. P = Present. NP = Not present. Table S2: Antibiotic resistance distribution among participants. S = sensitive. R = resistant.
