# Supplementary material for: Mobile Phones: Reservoirs of Resistant Bacteria during the COVID-19 Pandemic in Abu Dhabi, United Arab Emirates
Source: Microorganisms. 2023 Feb 18;11(2):523. doi: 10.3390/microorganisms11020523 (PMC9962086; doi:10.3390/microorganisms11020523)
Supplement: Supplementary file 1 [file microorganisms-11-00523-s001.zip › microorganisms-2210006-supplementary.pdf]

Supplementary Table S1. Identified bacteria on participants' mobile surfaces. P= Present. NP= Not present.

# of sample	*Staphylococcus aureus*	*Streptococcus*	*Enterococcus*	*bacillius*	*Micrococcous*	*Coagulase-negative staphylococci(CoNS)*	*E.coli*	*citrobacter*	*proteus*	*Klebsiella spp*	*pseudomonas aeruginosa*	*Actinobacter*
1	P	NP	NP	NP	NP	NP	NP	NP	NP	NP	NP	NP
2	P	NP	NP	NP	NP	NP	NP	NP	NP	NP	NP	NP
3	P	NP	NP	NP	NP	NP	NP	NP	NP	NP	NP	NP
4	NP	P	P	NP	NP	NP	P	NP	NP	NP	NP	NP
5	P	NP	NP	NP	NP	NP	NP	NP	NP	NP	NP	NP
6	P	NP	NP	NP	NP	NP	NP	P	P	NP	NP	NP
7	P	NP	NP	NP	NP	NP	P	NP	NP	NP	NP	NP
8	P	NP	NP	NP	NP	NP	NP	NP	NP	NP	NP	NP
9	P	NP	NP	NP	NP	NP	NP	NP	NP	NP	NP	NP
10	P	NP	NP	NP	NP	NP	NP	NP	NP	NP	NP	NP
11	P	NP	NP	NP	NP	NP	NP	NP	NP	NP	NP	NP
12	NP	NP	NP	NP	P	NP	NP	NP	NP	NP	NP	NP
13	P	NP	NP	NP	NP	NP	NP	NP	NP	NP	NP	NP
14	NP	NP	NP	NP	NP	P	NP	NP	NP	NP	NP	NP
15	NP	NP	NP	NP	P	NP	P	NP	NP	NP	NP	NP
16	NP	P	P	NP	NP	NP	NP	NP	NP	NP	NP	NP
17	NP	NP	NP	P	NP	NP	NP	NP	NP	NP	NP	NP
18	P	NP	NP	NP	NP	NP	NP	NP	NP	NP	NP	NP
19	P	NP	NP	NP	NP	NP	NP	NP	NP	NP	NP	NP
20	P	NP	NP	NP	NP	NP	NP	NP	NP	NP	NP	NP
21	NP	NP	NP	NP	P	NP	NP	NP	NP	P	P	P

22	NP	P	P	NP	NP	NP	NP	NP	NP	NP	NP	NP
23	NP	NP	NP	NP	P	NP	NP	P	P	NP	NP	NP
24	P	NP	NP	NP	NP	NP	NP	NP	NP	NP	NP	NP
25	P	NP	NP	P	NP	NP	P	NP	NP	NP	NP	NP
26	NP	NP	NP	NP	NP	P	NP	NP	NP	NP	NP	NP
27	NP	P	P	NP	NP	NP	P	NP	NP	NP	NP	NP
28	P	NP	NP	NP	NP	NP	NP	NP	NP	NP	NP	NP
29	P	NP	NP	NP	NP	NP	NP	NP	NP	NP	NP	NP
30	NP	P	P	NP	NP	NP	P	NP	NP	NP	NP	NP
31	P	NP	NP	P	NP	NP	NP	NP	NP	NP	NP	NP
32	NP	NP	NP	NP	NP	P	NP	NP	NP	NP	NP	NP
33	NP	NP	NP	NP	NP	P	P	NP	NP	NP	NP	NP
34	NP	NP	NP	NP	NP	P	NP	NP	NP	NP	NP	NP
35	NP	NP	NP	NP	NP	P	P	NP	NP	NP	NP	NP
36	P	NP	NP	NP	NP	NP	NP	NP	NP	NP	NP	NP
37	NP	NP	NP	NP	NP	P	NP	NP	NP	NP	NP	NP
38	P	NP	NP	P	NP	NP	NP	NP	NP	NP	NP	NP
39	NP	NP	NP	NP	P	NP	NP	NP	NP	NP	NP	NP
40	NP	P	P	NP	NP	NP	NP	NP	NP	NP	NP	NP
41	NP	NP	NP	NP	NP	P	P	NP	NP	NP	NP	NP
42	NP	NP	NP	NP	NP	P	NP	NP	NP	NP	NP	NP
43	NP	NP	NP	NP	NP	P	NP	NP	NP	P	P	P
44	NP	P	NP	NP	NP	NP	NP	NP	NP	NP	NP	NP
45	P	NP	NP	P	NP	NP	NP	NP	NP	NP	NP	NP
46	NP	NP	NP	NP	P	NP	NP	P	P	NP	NP	NP
47	NP	P	NP	NP	NP	NP	NP	NP	NP	NP	NP	NP
48	NP	NP	NP	NP	NP	P	NP	NP	NP	NP	NP	NP

49	NP	P	P	NP	NP	NP	P	NP	NP	NP	NP	NP
50	NP	NP	NP	NP	NP	P	NP	NP	NP	NP	NP	NP
51	NP	NP	NP	NP	NP	P	NP	NP	NP	NP	NP	NP
52	NP	NP	NP	NP	NP	P	NP	NP	NP	NP	NP	NP
53	P	NP	NP	P	NP	NP	NP	NP	NP	P	P	P
54	NP	NP	NP	NP	NP	P	NP	P	P	NP	NP	NP
55	NP	NP	NP	NP	NP	P	NP	NP	NP	NP	NP	NP
56	NP	NP	NP	NP	P	NP	NP	P	P	NP	NP	NP
57	NP	NP	NP	NP	NP	P	NP	NP	NP	P	NP	NP
58	NP	NP	NP	NP	P	NP	NP	P	P	NP	NP	NP
59	P	NP	NP	P	NP	NP	NP	P	P	NP	NP	NP
60	NP	P	NP	NP	NP	NP	NP	P	P	NP	NP	NP
61	NP	NP	NP	NP	NP	P	NP	NP	NP	NP	NP	NP
62	NP	NP	NP	NP	P	NP	NP	NP	NP	NP	NP	NP
63	P	NP	NP	NP	NP	NP	NP	NP	NP	NP	NP	NP
64	NP	NP	NP	NP	NP	P	NP	NP	NP	P	P	P
65	P	NP	NP	P	NP	NP	P	NP	NP	NP	NP	NP
66	NP	NP	NP	NP	P	NP	NP	NP	NP	NP	NP	NP
67	NP	NP	NP	NP	P	NP	P	NP	NP	NP	NP	NP
68	NP	P	NP	NP	NP	NP	P	NP	NP	NP	NP	NP
69	P	NP	NP	P	NP	NP	NP	NP	NP	NP	NP	NP
70	P	NP	NP	P	NP	NP	NP	P	P	NP	NP	NP
71	NP	NP	NP	NP	P	NP	NP	P	P	NP	NP	NP
72	P	NP	NP	P	NP	NP	NP	P	P	NP	NP	NP
73	NP	NP	NP	NP	NP	P	NP	NP	NP	NP	NP	NP
74	P	NP	NP	P	NP	NP	NP	NP	NP	NP	NP	NP
75	NP	NP	NP	NP	P	NP	NP	NP	NP	NP	NP	NP

76	P	NP	NP	P	NP	NP	NP	NP	NP	NP	NP	NP
77	P	NP	NP	NP	NP	NP	NP	NP	NP	NP	NP	NP
78	NP	P	NP	NP	NP	NP	P	NP	NP	NP	NP	NP
79	NP	P	NP	NP	NP	NP	NP	NP	NP	NP	NP	NP
80	NP	NP	NP	NP	P	NP	NP	NP	NP	NP	NP	NP
81	NP	NP	NP	NP	NP	P	NP	NP	NP	NP	NP	NP
82	P	NP	NP	NP	NP	NP	NP	NP	NP	NP	NP	NP
83	NP	NP	NP	NP	P	NP	P	NP	NP	NP	NP	NP
84	NP	NP	NP	NP	P	NP	P	NP	NP	NP	NP	NP
85	P	NP	NP	NP	NP	NP	NP	NP	NP	NP	NP	NP
86	NP	P	P	NP	NP	NP	NP	NP	NP	NP	NP	NP
87	P	NP	NP	NP	NP	NP	NP	NP	NP	NP	NP	NP
88	NP	NP	NP	NP	P	NP	NP	NP	NP	NP	NP	NP
89	NP	NP	NP	NP	P	NP	P	NP	NP	NP	NP	NP
90	NP	NP	NP	NP	P	NP	NP	NP	NP	NP	NP	P
91	NP	NP	NP	NP	P	NP	NP	NP	NP	NP	NP	P
92	NP	NP	NP	NP	NP	P	P	NP	NP	NP	NP	NP
93	NP	NP	NP	NP	P	NP	NP	NP	NP	NP	NP	NP
94	NP	NP	NP	NP	NP	P	NP	NP	NP	NP	NP	NP
95	P	NP	NP	P	NP	NP	NP	P	P	NP	NP	NP
96	NP	NP	NP	NP	P	NP	NP	P	P	NP	NP	NP
97	NP	NP	NP	NP	NP	P	NP	P	P	NP	NP	NP
98	NP	NP	NP	NP	P	NP	NP	NP	NP	NP	NP	NP
99	NP	NP	NP	NP	NP	NP	NP	NP	NP	NP	NP	NP
100	NP	NP	NP	P	NP	NP	NP	NP	NP	NP	NP	NP
101	NP	P	P	NP	NP	NP	P	NP	NP	NP	NP	NP
102	NP	P	P	NP	NP	NP	P	NP	NP	NP	NP	NP

Supplementary Table S2. Antibiotic resistance distribution among participants. S= sensitive. R= resistant.

# of sample	Ciprofloxacin	Cefotaxime	Ampicillin	Gentamicin	Ceftazidime
1	S	R	R	S	R
2	S	S	S	S	R
3	S	S	S	S	R
4	S	S	R	S	S
5	S	R	R	S	R
6	S	R	S	S	R
7	S	S	S	S	R
8	S	R	R	S	R
9	S	S	R	S	S
10	S	S	R	R	S
11	S	S	S	S	R
12	S	S	S	S	R
13	S	S	S	S	S
14	S	S	S	S	S
15	S	S	R	S	S
16	S	S	R	S	R
17	S	S	S	S	R
18	S	S	R	S	R
19	S	R	R	S	R
20	S	R	R	S	R
21	S	R	S	S	R
22	S	S	S	S	R
23	S	S	R	S	S
24	S	S	R	S	R

25	S	R	S	S	S
26	S	R	R	S	R
27	S	S	R	S	S
28	S	R	R	S	R
29	S	R	S	S	R
30	S	R	R	S	R
31	S	S	S	S	S
32	S	S	S	S	S
33	S	S	R	S	S
34	S	S	S	S	S
35	S	S	R	S	R
36	S	S	S	S	S
37	S	R	R	S	R
38	S	R	R	S	R
39	S	S	S	S	S
40	S	S	S	S	S
41	S	S	S	S	R
42	S	R	R	S	R
43	S	S	R	S	S
44	S	R	R	S	R
45	S	R	R	S	S
46	S	R	R	S	R
47	S	R	R	S	R
48	S	S	S	S	R
49	S	S	S	S	R
50	S	S	R	S	S
51	S	S	S	S	S
52	S	S	R	S	S
53	S	R	R	S	R

54	S	S	R	S	S
55	S	R	R	S	R
56	S	R	R	S	R
57	S	S	S	S	S
58	S	S	S	S	S
59	S	R	R	S	R
60	S	R	R	S	R
61	S	R	R	S	R
62	S	R	R	S	R
63	S	S	R	S	S
64	S	R	R	S	R
65	S	R	R	S	R
66	S	S	S	S	S
67	S	R	R	S	R
68	S	R	R	S	R
69	S	S	S	S	S
70	S	S	R	S	R
71	S	S	S	S	S
72	S	R	R	S	R
73	S	S	S	S	S
74	S	R	R	S	R
75	S	R	R	S	R
76	S	R	R	S	R
77	S	R	R	S	R
78	S	R	R	S	R
79	S	R	R	S	R
80	S	S	S	S	S
81	S	S	R	S	S
82	S	R	S	S	R

83	S	S	R	S	S
84	S	S	S	S	S
85	S	R	R	S	S
86	S	S	R	S	S
87	S	S	R	S	S
88	S	S	R	S	S
89	S	S	R	S	S
90	S	S	R	S	R
91	S	S	R	S	S
92	S	S	R	S	S
93	S	R	R	S	R
94	S	S	R	S	S
95	S	S	R	S	S
96	S	S	R	S	S
97	S	R	R	S	R
98	S	R	R	S	R
99	S	R	R	S	R
100	S	R	R	S	R
101	S	R	R	S	R
102	S	R	R	S	R
